# Effects of vibration therapy on muscle mass, muscle strength and physical function in older adults with sarcopenia: a systematic review and meta-analysis

**DOI:** 10.1186/s11556-020-00247-5

**Published:** 2020-09-17

**Authors:** Shuang Wu, Hong-Ting Ning, Su-Mei Xiao, Ming-Yue Hu, Xin-Yin Wu, Hong-Wen Deng, Hui Feng

**Affiliations:** 1 Xiangya school of nursing, Central South University, Changsha, Hunan province China; 2 Department of Public health, Sun Yat-Sen University, Guangzhou, Guangdong province China; 3 Department of Epidemiology and Biostatistics, Xiangya school of Public health, Central South University, Changsha, Hunan province China; 4 School of basic medical science, Central South University, Changsha, Hunan province China; 5 Xiangya-Oceanwide Health Management Research Institute, Central South University, Changsha, China

**Keywords:** Sarcopenia, Vibration therapy, Intervention, Muscle mass, Muscle strength, Physical performance

## Abstract

**Background:**

Sarcopenia, a progressive loss of muscle mass and function with advancing age, is a prevalent condition among older adults. As most older people are too frail to do intensive exercise and vibration therapy has low risk and ease of participation, it may be more readily accepted by elderly individuals. However, it remains unclear whether vibration therapy would be effective among older adults with sarcopenia. This systematic review and meta-analysis examined the effect of vibration therapy including local vibration therapy and whole-body vibration therapy, for enhancing muscle mass, muscle strength and physical function in older people with sarcopenia.

**Methods:**

A systematic literature search was conducted in March 2019 in the following 5 electronic databases: PubMed, CINAHL, Embase, PEDro, and the Cochrane Central Register of Controlled Trials, with no restriction of language or the year of publication. Randomized controlled trials and quasi-experimental studies examining effects of vibration therapy on muscle mass, muscle strength or physical function in older adults with sarcopenia were included in this systematic review. Two reviewers independently assessed the methodological quality of the selected studies.

**Results:**

Of the 1972 identified studies, seven publications from six studies involving 223 participants were included in this systematic review. Five of them conducted whole-body vibration therapy, while two conducted local vibration therapy. A meta-analysis of randomized controlled studies indicated that muscle strength significantly increased after whole-body vibration therapy (SMD 0.69, 95% CI 0.28 to 1.11, I^2^ = 0%, *P* = 0.001) and local vibration therapy (SMD 3.78, 95% CI 2.29 to 5.28, *P* < 0.001). Physical performance measured by the sit-to-stand test and the timed-up-and-go test were significantly improved after the intervention (SMD -0.79, 95% CI − 1.21 to − 0.37, I^2^ = 0%, *P* < 0.001) and SMD -0.83, 95% CI − 1.56 to − 0.11, I^2^ = 64%, *P* = 0.02, respectively).

**Conclusion:**

Vibration therapy could be a prospective strategy for improving muscle strength and physical performance in older adults with sarcopenia. However, due to the limited number of the included studies, caution is needed when interpreting these results. More well-designed, large sample size studies should be conducted to further explore and validate the benefits of vibration therapy for this population.

## Introduction

The ageing population is rapidly increasing worldwide [1]. The ageing process is responsible for marked changes in multiple tissues and organs, especially for skeletal muscle [2]. From age 40 to 80, total skeletal muscle mass declines 30 to 50% in both men and women [3, 4]. The term sarcopenia was first coined by Rosenberg et al. in 1989 as the progressive loss of muscle mass with advancing age [5]. With less muscle mass, muscle strength and muscle function are greatly reduced [6]. However, the loss of muscle strength is much more rapid than the parallel loss of muscle mass [7]. Subsequently, sarcopenia has been defined as a deficiency in muscle mass plus decreased muscle strength or the impaired physical performance [6].

Sarcopenia is prevalent in the older population. It impairs personal health and reduces life quality, while at the same time putting a heavy financial burden on the healthcare system [8]. A recent systematic review found that the prevalence of sarcopenia was 1–29% in the community, 14–33% in long-term care institutions and 10% in hospitals [9]. Sarcopenia results in a higher risk of disability, depression, mortality, increasing the risk of fall-related injury and the possibility of being admitted to a long-term care facility [10–13]. The health care cost of sarcopenia in the United States alone was estimated at $18.5 billion or approximately 1.5% of total healthcare expenditure in the year 2000 [14, 15]. Additionally, sarcopenia will increase hospitalization costs by 34% among patients 65 years and older [16]. Early intervention is the key point to improve the outcomes in older adults with sarcopenia.

The most effective physiologic way to prevent and treat sarcopenia and related muscle malfunction is a physically active lifestyle, or even better, physical exercise [17, 18]. For example, aerobics, endurance exercise and resistance exercise training have been regarded as the main strategies for preventing physical function decline [19]. However, these conventional exercises may not be suitable for weak individuals (i.e., aged or frail individuals or elderly individuals with physical limitations), especially institutionalized elderly persons, as their muscle strength can deteriorate to a point where it becomes critical for independence during transfers and walking [20–22].

An alternative to traditional exercise technology is vibration therapy (VT). It may be a safe, autonomous, and efficient way to increase or maintain muscle mass, strength and function for elderly and weak individuals, who are unable or unwilling to perform conventional workouts [23–27]. When VT was added to conventional resistive exercise, a great improvement in muscle power was reported [28]. VT is a training modality that uses mechanical oscillations as a stimulus for human neuromuscular structures, where the energy is transferred from the vibration device to the human body or parts of it [28–30]. The mechanical stimulus produced is thought to use proprioceptive spinal reflexes to increase muscle function by enhancing muscle spindle excitatory signaling while lowering the inhibitory response of the Golgi tendon organ to the motoneuron pool [28, 31]. Similar to the effects of resistance training and plyometric training, vibration stimulus increases the gravitational load on the neuromuscular system, thereby providing a stimulus that modifies the functional capacity of skeletal muscle [32]. VT can be applied to the targeted muscles mainly by two ways: whole-body vibration (participants squat or stand on the vibrating platforms) and local vibration (applied superficially over the targeted muscle) [20, 33].

A growing number of clinical trials have demonstrated the favourable impacts of VT on postural control [34], mobility [35–37], lean body mass, muscle strength and physical performance [38–40], quality of life [41], efficacy and safety [42] in healthy elderly individuals. However, when trials were conducted on frail elderly individuals, hospitalized elderly individuals or more specifically, older adults with sarcopenia, improvement in muscle function was not reported [43, 44].

Up to now, some systematic reviews [9, 43, 45] have synthesized the evidence of physical activity in sarcopenia, none of which have included VT. No systematic review has synthesized the evidence of VT among elderly individuals with sarcopenia [20]. Therefore, it is now necessary to conduct a systematic review to examine all the evidence and clarify the effects of VT on muscle mass, muscle strength and physical performance in elderly patients with sarcopenia. The findings from this study could be used to guide clinical decision-making in interventions and treatments for sarcopenia.

## Methods

### Protocol and registration

This systematic review was registered in the PROSPERO international prospective register of systematic reviews (no. CRD42019128866). We followed the Preferred Reporting Items for Systematic review and Meta-Analysis (PRISMA) guidelines in conducting this review [46].

### Eligibility criteria

Abstract-only studies and reports were excluded from this review because of the limited information on the intervention and participants’ characteristics, as well as the difficulty of determining the specific quality of these studies

The inclusion criteria were as follows: 1) Randomized control studies or quasi-experimental studies; 2) The studies should have clear and detailed diagnostic criteria for sarcopenia, no matter which one was used in the study; 3) VT (local VT or whole-body VT) was used in the study, regardless of type; 4) Outcomes of the studies included at least one of the following data results: muscle mass, muscle strength or physical function. Studies were excluded if they included individuals who had evidence of hereditary or acquired muscular disease or were under treatment with testosterone or other pharmacological interventions known to influence muscle mass or if they lacked related outcomes.

### Search strategy

A systematic literature search was conducted in March 2019 in the following 5 electronic databases: PubMed, CINAHL, Embase, PEDro, and the Cochrane Central Register of Controlled Trials, with no restriction of language or year of publication. The search terms used were as follows: (sarcopeni* OR muscular atrophy OR muscle weakness OR muscle mass OR fat free mass OR lean body mass OR lean mass OR body composition OR hand strength OR grip strength) AND (aged OR aging OR seniors OR elderly OR older) AND (vibration OR whole body vibration OR whole body vibration training OR vibration exercise OR vibration platform OR vibratory therapy OR vibratory plate OR sham therapy OR Wbv OR low intensity vibration OR LIV OR VbX OR WBVT). The exact search syntaxes used are listed in Appendix 1 (Supplementary Table 1). Reference lists of relevant articles were also manually searched, and authors were conducted for additional data, if necessary, for the systematic review.

### Study selection

Two reviewers independently assessed potential eligible studies by screening the titles, abstracts, and full texts. In case of disagreement, consensus was sought between the reviewers, or a third reviewer was asked. Duplicates were identified and excluded, and multiple articles of the same study were collated so that each study, rather than each article, was the unit of interest in the review.

### Data extraction and quality assessment

Data extraction was performed by two reviewers independently using a standardized data collection form that included the year of publication, first author, subjects and their sex, age of participants, study design, diagnostic criteria for sarcopenia, settings, main outcomes and training protocols. All outcomes were reported as in the original articles.

Two reviewers independently assessed the selected randomized controlled trials according to the Risk of Bias Tool found in the Cochrane Handbook for Systemic Reviews of Interventions [47], with the following aspects: sequence generation and concealment of allocation (selection bias), blinding of participants and personnel (performance bias), blinding of outcome assessors (detection bias), incomplete outcome data addressed (attrition bias), free of selective reporting (reporting bias), and other bias. The quality of non-randomized studies was assessed using the Methodological Index for Non-Randomized Studies (MINORS) tool [48]. The MINORS tool identifies 12 items, including 8 specifically designed for non-comparison studies: a clearly stated aim, the inclusion of consecutive patients, a prospective collection of data, endpoints appropriate to the aim of the study, an unbiased assessment of the study endpoints, a follow-up period appropriate to the aim of the study, loss to follow-up less than 5%, and a prospective calculation of the study size. The items are scored as follows: 0 = reported, 1 = reported but inadequate, and 2 = reported and adequate. Results for non-comparison studies range from 0 (low quality) to 16 (high quality). Disagreement on the quality rating between the reviewers was settled by discussing or consulting with the senior researchers if necessary.

### Data synthesis and analysis

We followed the Cochrane Handbook for Systematic Results of Interventions to handle and analyse the data to run the meta-analysis [49]. Outcomes are presented as the mean change from baseline in muscle mass, muscle strength and physical performance. All outcomes are continuous variables. The meta-analysis was conducted using Review Manager, version 5.3 (Cochrane, London, UK). In the meta-analyses, standard mean differences (SMD) and 95% confidence intervals (CIs) were used for continuous data. The results were regarded as statistically significant when *P* < 0.05. Heterogeneity across studies was tested by using the I^2^ statistic, which is a quantitative measure of inconsistency across studies. Studies were considered to have low heterogeneity when the I^2^ statistic was 25–50%, and those with an I^2^ statistic > 75% were considered to have high heterogeneity. A random-effects model was used if the I^2^ statistic > 50%, otherwise the fixed-effect model was used [50].

## Results

### Study selection

**Fig. 1:**
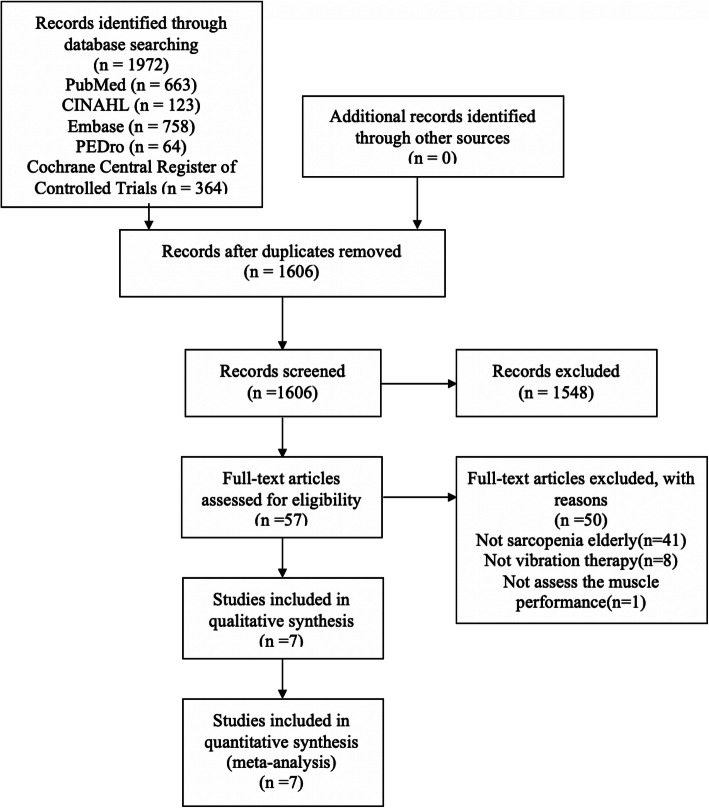
Flowchart showing how the reviewed articles were identified and selected

A PRISMA flowchart of the literature search and study selection are demonstrated in Fig. 1 [46]. We identified a total of 1972 records, with 1606 records left after duplicates were removed. Then, 1548 records were excluded after screening the title and abstract, leaving 57 articles for full-text review. Among the 57 articles, 50 were excluded due to the following reasons: not focused on sarcopenia in an elderly population (*n* = 41), not VT (*n* = 8), no assessment of muscle performance (*n* = 1). The remaining 7 articles were assessed for methodological quality. All 7 articles were considered to have met the quality standards of methodology and were retained for the systematic review. The seven articles [51–57] came from six clinical studies, so six research groups were included in this systematic review. Studies with two publications [54, 55] were considered as a single study throughout the systematic review.

### Quality of the study

**Fig. 2:**
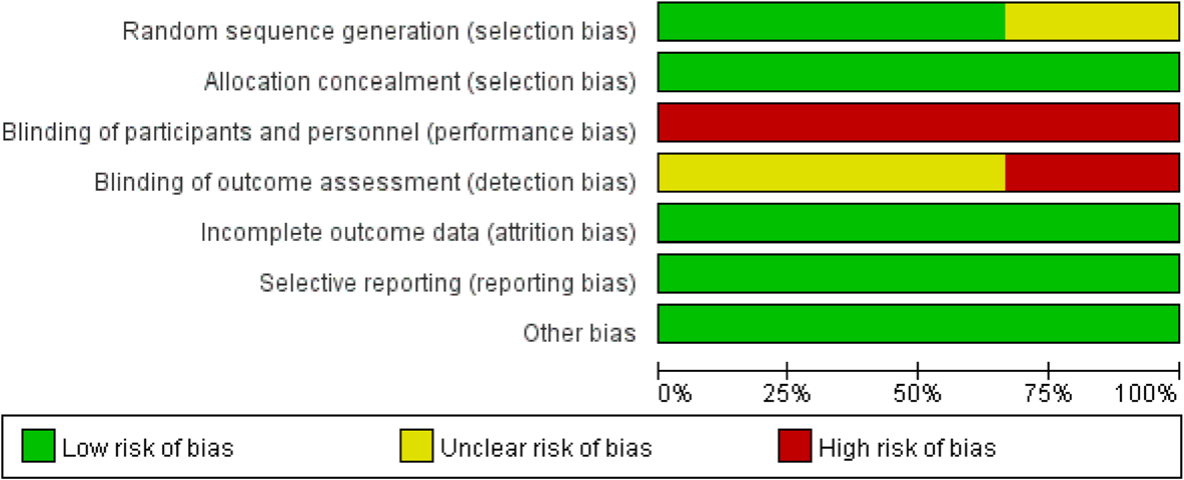
Risk of Randomized control studies bias graph

**Fig. 3:**
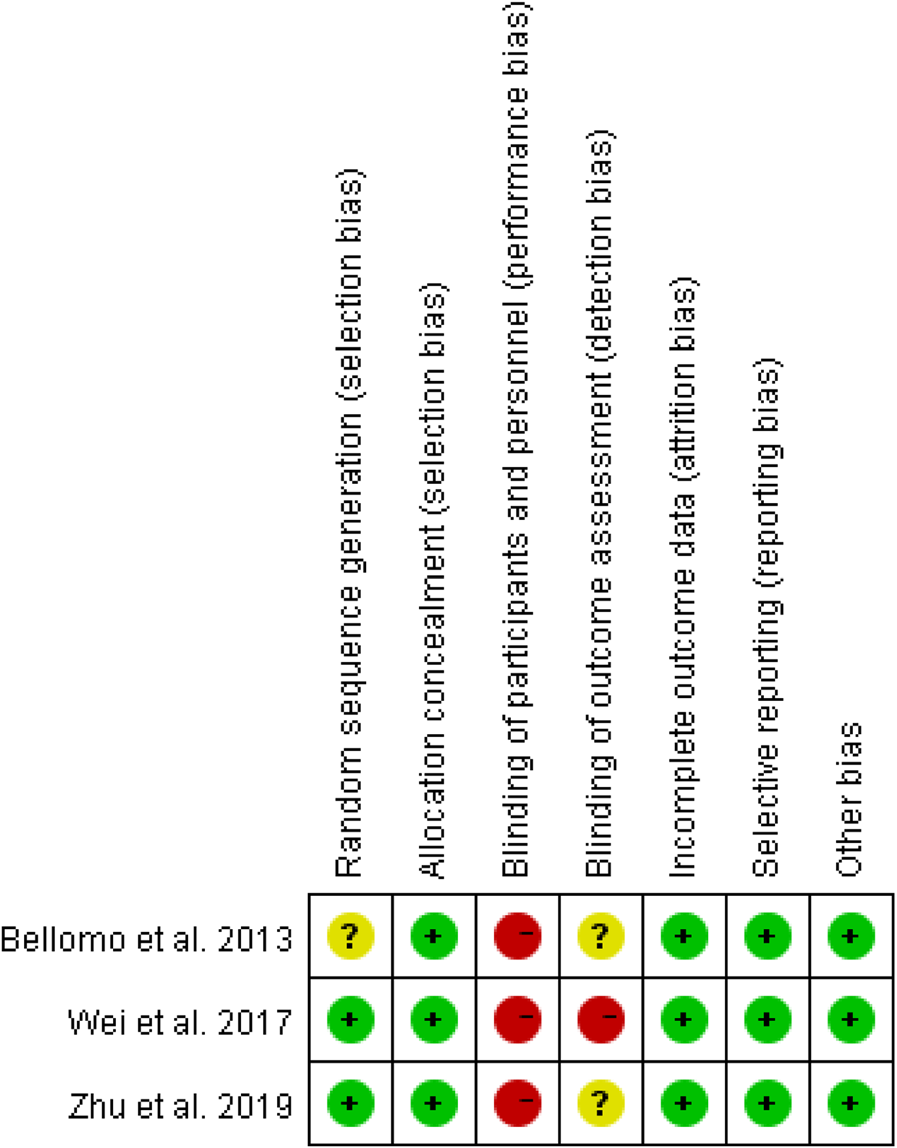
Risk of Randomized control studies bias summary

The results of the quality assessment using the Cochrane Collaboration Recommendations assessment tools are reported in Fig. 2 and Fig. 3, and those using the MINORS tool are reported in Supplementary Table 2. The quality of the included studies were found to be acceptable. The three randomized controlled trials showed a high risk of performance bias, which is inevitable. The MINORS scores ranged from 11 to 13 out of a possible 16 for the quasi-experimental studies. Most studies received a score of 0 for the item for unbiased assessment of the study endpoint. Due to the VT, it was not possible to blind patients or study personnel to the group allocation.

### Study characteristics

The 6 clinical studies, composed of 3 quasi-experimental studies and 3 randomized controlled trails, involved 208 participants with sarcopenia. All of them included subjects aged 60 years or older, except one study [51] (58.2 ± 6.4 years old). The number of participants ranged from 9 to 80. The diagnostic criteria of sarcopenia among these studies were different, and only one study [57] published in 2019 used the diagnostic criteria of sarcopenia from the Asian Working Group for Sarcopenia (AWGS). The others used the diagnostic criteria from a variety of previous studies. Details are shown in Table 1.

**Table 1:** The characteristics of participants included in the review

Study year	Study design	Country	Subjects number T (male, female) C (male, female)	Mean age	Diagnosing criteria of sarcopenia
Bellomo et al. 2013 [53]	RCT	American	T:10(10,0) C:10(10,0)	70.9 ± 5.2	SMI (kg/m^2^) by DXA < 2 SD of a young reference group
Wei et al. 2017 [54, 55]	RCT	China (Hong Kong)	T1:20(7,13) T2:20(7,13) T3:20(5,15) C:20(5,15)	T1:78 ± 4 T2:75 ± 6 T3:74 ± 5 C:76 ± 6	SMI (kg/m^2^) by BIA, cutoff 8.87 kg/m^2^ for male, cutoff 6.42 kg/m^2^ for female
Zhu et al. 2019 [57]	RCT	Mainland of China	T:28(NR) C:27(NR)	T:89.5 ± 4.4 C:87.5 ± 3	SMI (kg/m^2^) by DXA cutoff values 7.0 kg/m^2^ for male and 5.4 kg/m^2^ for female; grip strength cutoff 26 kg for male and 18 kg for females, walking speed cutoff value 0.8 m/s from AWGS
Pietrangelo et al. 2009 [52]	CCT	Italy	T:9(4,5)	M:75.3 ± 6.9 F:71.0 ± 5.7	SMI (kg/m^2^) by DXA < 2 SD of a young reference group
Chang et al. 2018 [56]	CCT	China (Taiwan)	17(12,5)	82.12 ± 8.19	SMI (kg/kg) by body composition analyzer (model IOI353) cutoff for males 10.75 kg/m^2^, and females 6.75 kg/m^2^ grip strength cutoff 26 kg for male and 18 kg for females, walking speed cutoff value 0.8 m/s Standards
Miller et al. 2018 [51]	CCT	American	15(0/15)	58.2 ± 6.4	ALM/BMI cutoff values 0.789 for male and 0.512 for female

*SMI* (kg/m^2^): muscle mass (kg)/height (m)^2^, *SMI* (kg/kg): muscle mass (kg)/weight(kg), ALM/BMI: ALM (appendicular lean mass)/BMI (body mass index), *DXA* Dual energy X-ray absorptiometry, *BIA* Bioimpedance analysis, *AWGS* Asian Working Group for Sarcopenia

The characteristics of the VT protocols and outcome measurements are outlined in Table 2. Overall, 145 participants received VT. Two studies [52, 53], including 19 participants investigated local VT, with a vibration frequency of 300 Hz. The other 4 studies [51, 54–57], including 126 participants, adopted whole-body VT, and the vibration parameters were different among these studies, varying from 12 [56, 57] to 60 [54, 55] Hz. The postures of participants on the whole-body VT platform were standing [56], half-squat standing [51, 54, 55], and sitting [57]. The training programmes in these 4 studies [52–57] were long-term programmes, and the duration of all programmes was 12 weeks, except for one [57] that was 8 weeks. The remaining study [51] measured the acute effects of whole-body VT. All the studies [51–53, 56, 57] measured the relevant phenotypes before and after the intervention, and two studies [52, 54, 55] continued follow-up after the intervention for 12 weeks.

**Table 2:** The characteristics of training protocol and outcomes included in the review

Study year	Type of intervention (T/C)	Vibration machine	Posture of WBV or location of LV	Vibration Frequency (Hz)	Time of duration	Frequency of sessions ×duration of program	Outcome measures and (follow up period in weeks)		
							Muscle mass	Muscle strength	Physical performance
Bellomo et al. 2013 [53]	T:LV C:None	VISS device (Vissman, Rome, Italy)	Vastus medialis, vastus lateralis and rectus femoris muscles	300	15 min	1/week from week 1 to 8 3/week from week 9 to 12	NM	Lower-limb strength (0,12)	Balance test: Sway area (0,12)
Wei et al. 2017 [54, 55]	T1: WBV T2: WBV T3: WBV C: None	WBV machine (Fit vibe excel, Gymna Uniphy NV, Bilzen, Belgium)	Stood barefoot with their knee joint flexed at 60° on the platform of the WBV machine with hands holding onto the rail in front	T1:20 T2:40 T3:60	12 min 6 min 4 min	3/week from 1 to 12	CSA (0,6,12,18,24)	Lower-limb strength (0,6,12,18,24)	Meter walking test Timed up and-go test Five-repetition sit-to-stand test (0,6,12,18,24)
Study year	Type of intervention (T/C)	Vibration machine	Posture of WBV or location of LV	Vibration Frequency (Hz)	Time of duration	Frequency of sessions ×duration of program	Outcome measures and (follow up period in weeks)		
							Muscle mass	Muscle strength	Physical performance
Zhu et al. 2019 [57]	T: WBV C: None	WBV machine Wellengang Excellence reciprocating vibration platform (SVG, Wellengang, Germany)	Set on the chair next to the WBV machine and put the foot on the fixed position of the WBV machine with hands holding onto the rail in front	12 for 1 to 2 weeks 14 for 3 to 6 weeks 16 for 7 to 8 weeks	20 min	5/week from 1 to 8	DXA (0,8)	Handgrip strength Lower-limb strength (0,8)	The 6-m gait speed test Timed-up-and-go test Five-times-sit-to-stand test Balance test (0,8)
Pietrangelo et al. 2009 [52]	LV	VISS device (Vissman, Rome, Italy)	Intermedius femoris, rectus femoris, vastus medialis, and vastus lateralis muscles.	300	15 min	1/week from week 1 to 8 3/week from week 9 to 12	CSA (0,4,8,12)	Lower-limb strength (0,4,8,12,28)	NM
Study year	Type of intervention (T/C)	Vibration machine	Posture of WBV or location of LV	Vibration Frequency (Hz)	Time of duration	Frequency of sessions ×duration of program	Outcome measures and (follow up period in weeks)		
							Muscle mass	Muscle strength	Physical performance
Chang et al. 2018 [56]	WBV	Whole-body vibration (i-vib6050 model)	Stood on a vibration and stimulation generating platform	12	10 min	3/week from week 1 to week 12	SMI (kg/kg) (1, 12)	Grip strength (1, 12)	Eight-foot up and go test Five repeated sit-to-stand tests Standing on one-foot text Shoulder-arm flexibility text (1, 12)
Miller et al. 2018 [51]	WBV	Whole-body vibration (Power Plate platform (Northbrook, Illinois)	Stood bare foot on the platform, legs shoulder width apart, knees flexed to a 30° angle, and their arms placed equidistant on the device handles.	30	T1:6 min T2:1min	T1:1 time T2:6 time	NM	Grip strength (acute)	Timed Up and Go Test Berg Balance Scale Sit and Reach (acute)

*WBV* whole-body vibration, *LV* local vibration, *CSA* cross-sectional area, *SMI* (kg/kg): muscle mass (kg)/weight(kg), *DXA* Dual energy X-ray absorptiometry, *NM* not measured

### Impacts of different vibration intervention strategies

**Fig. 4:**
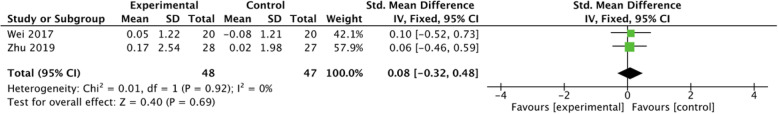
The forest plot of effect sizes of whole-body vibration therapy compared to control on muscle mass. Values on x-axis denote standardized mean differences. The diamond illustrates the 95% confidence interval of the pooled effects

Four studies assessed the effects of VT on muscle mass, including two randomized controlled trials [55, 57]. However, different measurement tools had been used in these studies: two of them [52, 55] used the cross-sectional area, one [56] used the weight-adjusted muscle mass index measured by bioelectrical impedance analysis, and one [57] adopted muscle mass measured by dual-energy X-ray absorptiometry. There was no significant increase in muscle mass in the studies by Wei et al. [55], Pietrangelo et al. [52] and Zhu et al. [57]. However, the study [56] that used the following weight-adjusted muscle mass formula: total skeletal muscle mass (in kg)/body weight (in kg) × 100, found that, after a 12-week intervention of whole-body VT, the muscle mass was significantly higher than that before the whole-body VT intervention. The two randomized controlled trials indicated that muscle mass did not show significant differences between the two groups (SMD 0.08, 95% CI − 0.32 to 0.48, I^2^ = 0%, *P* = 0.69) (Fig. 4).

**Fig. 5:**
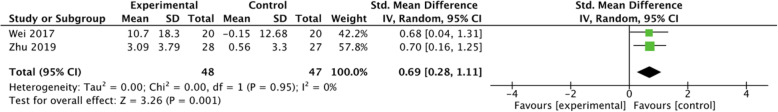
The forest plot of effect sizes of whole-body vibration therapy compared to control on muscle strength. Values on x-axis denote standardized mean differences. The diamond illustrates the 95% confidence interval of the pooled effects

**Fig. 6:**
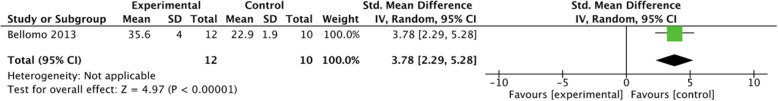
The forest plot of effect sizes of local vibration therapy compared to control on muscle strength. Values on x-axis denote mean differences. The diamond illustrates the 95% confidence interval of the pooled effects

Muscle strength was measured in all studies. Four studies [52, 53, 55, 57] used lower limb strength measurements as an indicator of muscle strength, while three studies [51, 56, 57] used grip strength. All the studies demonstrated that muscle strength increased significantly after the VT intervention, regardless of which indicators were used. The two randomized controlled trials using whole-body VT indicated that muscle strength showed a significant increase after whole-body VT (SMD 0.69, 95% CI 0.28 to 1.11, I^2^ = 0%, *P* = 0.001) (Fig. 5). One randomized controlled trial using local VT also indicated a significant increase in muscle strength after local VT (SMD 3.78, 95% CI 2.29 to 5.28, *P* < 0.001) (Fig. 6).

**Fig. 7:**
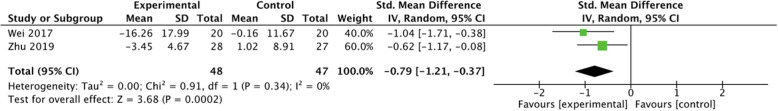
The forest plot of effect sizes of whole-body vibration therapy compared to control on five-repetition sit-to-stand test. Values on x-axis denote standardized mean differences. The diamond illustrates the 95% confidence interval of the pooled effects

**Fig. 8:**
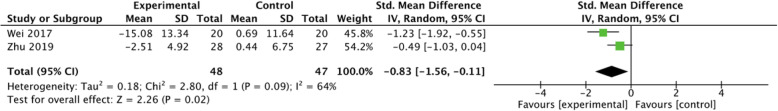
The forest plot of effect sizes of whole-body vibration therapy compared to control on timed-up-and-go test. Values on x-axis denote standardized mean differences. The diamond illustrates the 95% confidence interval of the pooled effects

Physical performance was measured in five studies. Different indicators were used among these studies. The most commonly used one was the timed up-and-go test, a coordination and agility test for elderly individuals, which was used in four studies [51, 54, 56, 57]. The findings of the four studies all revealed that the performance of the timed up-and-go test improved significantly after the whole-body VT. Three studies [54, 56, 57] used five repeated sit-to-stand tests as one of the indicators of physical performance, and all the findings showed that the time for five repeated sit-to-stand tests shortened significantly after the whole-body VT. Two studies [54, 57] adopted walking speed as one of the indicators of physical performance, and both studies suggested that walking speed improved significantly after whole-body VT. Balance tests were performed as one of the phenotypes of physical performance in 3 studies [51, 53, 57]. The findings of Miller et al. [51], using the Berg balance scale to assess static and dynamic balance capabilities, showed no significant improvement after the whole-body vibration intervention. The results of Bellomo et al. [53] demonstrated a significant improvement in the sway area and in the ellipse surface with open and closed eyes after 12 weeks of local VT. In a study by Zhu et al. [57], no significant differences were noted in static and dynamic balance capacity after 4 weeks of WBV exercise; however, significant improvements were observed after 8 weeks. Two randomized controlled trials that used whole-body VT indicated that the time for five repetitions of the sit-to-stand test and timed up-and-go test were significantly decreased after the intervention [(SMD -0.79, 95% CI − 1.21 to − 0.37, I^2^ = 0%, *P* < 0.001) (Fig. 7) and (SMD -0.83, 95% CI − 1.56 to − 0.11, I^2^ = 64%, *P* = 0.02) (Fig. 8), respectively].

## Discussion

Overall, this systematic review with the six currently available studies showed that VT may not have a notable effect on muscle mass compared to no treatment, but it has a significant impact on muscle strength and physical function in older adults with sarcopenia.

In this study, the eligible investigations were limited to VT, 2 [52, 53] for local VT and 5 [51, 54–57] for whole-body VT, and muscle mass, muscle strength or physical performance in older people with sarcopenia. Among them, different methods were used to diagnose sarcopenia, which could result in different severities of sarcopenia among the participants in the studies, thus increasing the risk of information bias. The interest in sarcopenia has risen in recent years, while universally accepted diagnostic criteria is still lacking [58]. Prior studies also mentioned this inevitable bias [44, 45]. Therefore, we recommend that future research should unify the diagnostic methods according to the consensus from the International Working Group on Sarcopenia [7], the European Working Group on Sarcopenia in Older People [59] and the Asian Working Group for Sarcopenia [60] to improve homogeneity in study populations and contribute to the understanding of the results. The vibration protocols were quite heterogeneous in terms of vibration type, vibration frequency, and vibration duration. It is hard for us to draw a conclusion as to the optimal vibration protocol. In previous studies, various vibration frequencies for whole-body VT have been used in frail populations [25, 61–63]. They used VT mainly between 12 and 30 Hz. We found whole-body VT uses a different vibration frequency and duration, which was mainly between 12 and 40 Hz and 1–20 min, respectively, than local VT, for which two studies used the same vibration frequency and duration of 300 Hz and 15 min, respectively.

Studies included in this systematic review reported that muscle mass might not increase significantly after VT, either whole-body VT or local VT. Only one included quasi-experimental study [56] found that the weight-adjusted muscle mass index (muscle mass/body weight) was improved after whole-body VT. However, this weight-adjusted muscle mass index ignored the change in body weight after whole-body VT. Studies have proved that whole-body VT could lead to weight loss [64, 65]. Therefore, the improvement in the weight-adjusted muscle mass index in this study may not be caused only by the change of muscle mass. Our findings suggested that VT did not provide sufficient stimulus for skeletal muscle hypertrophy in older adults with sarcopenia. These findings were in accordance with the systematic reviews performed by Chen et al. [66] and Beaudart et al. [43]. All of their results showed no impact of VT on muscle mass in regard to frail participants, participants residing in a nursing home or participants with limited mobility. The above participants may be too weak to tolerate a large dose of VT [9]. Furthermore, a small dose of VT would be difficult for older adults with sarcopenia who are inclined to lose muscle mass and who have a limited number of muscle spindles to excite to cause a significant increase in muscle mass [55]. However, we observed studies reporting that muscle mass increases ranged from 3.4 to 8.7% after whole-body VT [67, 68]. These studies required participants to exercise on the vibration platform, for instance, to perform a squat, deep squat, wide stance squat, toes-stand, deep toes-stand and one-legged squat. Standing position during WBV may facilitate the human response to a vibration stimulus [69, 70]. All the participants in this systematic review stood [56], half-squat stood [51, 54, 55] or sat [57] on the machine during the VT, which might remove the influence of exercise and thus not be sufficient to stimulate muscle hypertrophy in the participants.

A favourable impact of whole-body VT [69, 71, 72] and local VT [38, 73] on muscle strength was proposed by previous studies in healthy adults. This study found that it may also work in older adults with sarcopenia. According to the limited information we found, lower limb muscle strength increased from 38.4 to 41.7% [55, 57] during the intervention period with whole-body VT, but the increase could not be maintained after cessation of training. However, lower limb muscle strength increased by 43% after local VT, and the increase in strength was consistently maintained after local VT was interrupted for 12 weeks [53]. The reason could be that, during whole-body VT, the vibration energy was reduced by the activity of muscles in the lower extremity and the positions of the knee and ankle joints, which may influence the magnitude of the stimulus applied to proximal structures [74, 75]. Furthermore, the reduction of energy from VT could decrease if it was applied directly at the location of the muscle [76]. The reason could also be that whole-body VT was generally conducted with a lower vibration frequency and less duration time (vibration frequency varied from 12 to 60 Hz, and duration time varied from 1 to 20 min) than local vibration (vibration frequency was 300 Hz, and duration time was 15 min) in the included studies. Some frail participants cannot tolerate a high dose of whole-body VT, because they have difficulty squatting or standing on the vibrating platform for a long time or with a high vibration frequency. This situation also limits the effectiveness of whole-body VT [75]. However, we did not find any investigations that compared the effects of whole-body VT and local VT in older adults with sarcopenia. Further studies are needed to reveal whether local VT could be a more beneficial therapy for frail elderly individuals with sarcopenia.

Different senior physical fitness tests were adopted to assess physical performance in older adults with sarcopenia. This systematic review found that the timed up-and-go test and five repeated sit-to-stand tests were commonly used as physical performance measurements and were significantly improved after whole-body VT. These results were consistent with a series of systematic reviews aimed at the healthy elderly persons [34, 36, 42]. These two tests were not measured in the studies conducted by Bellomo et al. [53] and Pietrangelo et al. [52], which used local VT. Thus, we could not discern whether local VT had the same effect as the whole-body VT. However, for the balance test, which was measured after local VT and whole-body VT, both showed a favourable impact on the balance test. This might mean that VT can benefit the elderly individuals with sarcopenia with regard to balance function, just like for the healthy and frail elderly populations [35, 36, 77–80]. Disordered balance is the most common cause of falls in older adults and often leads to injury, disability, loss of independence, and limitations in quality of life [80, 81]. Appropriate interventions, such as VT, might prevent dysfunction or loss of independence.

Above all, VT did not significantly improve muscle mass. However, muscle strength and physical performance promoted compared with no treatment. Our findings, which reported that the increase in muscle strength was not in line with the changes in muscle mass, were similar to other studies [31, 70, 73]. Considering the non-parallel relationship between the training-induced changes in muscle mass and muscle strength, a possible reason could be that the mechanism of VT, namely, neuromuscular adaptations, caused an increase in type II fibres in the participants, and synchronization of motor units improved, rather than increased, in lean muscle mass [31, 73]. In addition, studies have suggested that the loss of muscle strength is more rapid than the loss of muscle mass in older adults and that the decline in the age-dependent strength cannot be explained by the loss of muscle mass alone [39, 82]. It has been shown that better muscle strength and physical performance are more vulnerable to the ageing process than muscle mass [83]. Therefore, VT that can improve muscle strength and physical performance is critical for older adults with sarcopenia, which is associated with better ability to perform daily life activities and mobility, improvement in quality of life and reduced healthcare costs [72, 84].

Some strengths of this systematic review should also be highlighted. We searched 5 electronic databases with no restriction on language or the year of publication. In addition to this, we also manually searched the references of the included studies for a broader research. To our knowledge, this is the first systematic review that included all the studies available to describe the effects of VT in older adults with sarcopenia according to the suggestion of the guidelines. However, our findings need to be interpreted with caution due to the potential limitations. First, only six studies with three randomized controlled trials were included. We could not draw more certain conclusions based on the small number of randomized controlled trials. Apart from this, the overall methodological quality of the included studies ranged from moderate to excellent; among these, two studies were high quality, and the others were moderate. Third, the diagnostic criteria, vibration protocols and outcome measures that used in the included studies were distinct, making direct comparison difficult, and the high heterogeneity in the meta-analysis results could have led to an overestimation of the effects.

### Implications for future research

Although this systematic review provided evidence that VT had positive effects on older adults with sarcopenia, we should consider that there was great variety among the studies concerning sample size, degree of sarcopenia, types of interventions and types of assessments. In addition, the absence of changes in some of the outcomes explored in this analysis indicated that VT must be carefully adapted to the sample of older adults with sarcopenia. Moreover, as the included studies only compared one type of VT with no treatment, we do not know the effectiveness of VT compared with conventional exercises or among the different types of VT. These limitations suggest that further research is needed to unify diagnostic methods according to consensus and compare different vibration types (local vibration and whole-body vibration), vibration times, vibration frequencies, vibration amplitudes and vibration magnitudes in elderly individuals with sarcopenia. More in-depth research comparing VT with other exercises that have been proven effective is needed.

## Conclusions

Compared with no treatment, VT showed the potential to provide positive benefits in improving the muscle strength and physical performance of older adults with sarcopenia. However, no significant improvement was found in terms of muscle mass. Due to inherent imprecision (limited sample size of the participants) and publication bias (the number of studies included was less than 10 for each outcome), the level of evidence was downgraded. To apply VT in elderly individuals with sarcopenia, more well-designed, large sample size randomized controlled clinical trials are needed to examine efficacy and different regimens.

## Supplementary information

**Additional file 1 Table S1.** Search strategy. **Table S2.** MINORS scores of quasi-experimental studies.

## Data Availability

All data generated or analysed during this study are included in this published article [and its supplementary information files].

## Abbreviation

VT: Vibration therapy
