# Supplementary material for: Effects of vibration therapy on muscle mass, muscle strength and physical function in older adults with sarcopenia: a systematic review and meta-analysis
Source: Eur Rev Aging Phys Act. 2020 Sep 17;17:14. doi: 10.1186/s11556-020-00247-5 (PMC7499918; doi:10.1186/s11556-020-00247-5)
Supplement: Supplementary file 1 — Additional file 1 Table S1. Search strategy. Table S2. MINORS scores of quasi-experimental studies. [file 11556_2020_247_MOESM1_ESM.docx]

Supplementary Table 1: Search strategy

| PubMed | | |
| --- | --- | --- |
| #1 | sarcopenia [MeSH Terms] OR muscular atrophy [MeSH Terms] OR muscle weakness[MeSH Terms] | 27677 |
| #2 | sarcopeni*[Title/Abstract] OR muscular atrophy[Title/Abstract] OR muscle weakness[Title/Abstract] OR fat free mass[Title/Abstract] OR lean body mass[Title/Abstract] OR lean mass[Title/Abstract] OR body composition[Title/Abstract] OR grip strength[Title/Abstract] OR physical function[Title/Abstract] OR Anthropometry[Title/Abstract] | 93225 |
| #3 | #1 OR #2 | 113499 |
| #4 | aged [MeSH Terms] OR aging [MeSH Terms] OR middle aged[MeSH Terms] | 4870778 |
| #5 | seniors [Title/Abstract] OR elderly [Title/Abstract] OR older[Title/Abstract] OR aged[Title/Abstract] OR aging[Title/Abstract] | 1082018 |
| #6 | #4 OR #5 | 5338631 |
| #7 | vibration [MeSH Terms] | 23959 |
| #8 | vibration[All Fields] OR whole body vibration[All Fields] OR whole body vibration training[All Fields] OR vibration exercise[All Fields] OR vibration platform[All Fields] OR vibratory therapy[All Fields] OR vibratory Plate[All Fields] OR sham therapy[All Fields] OR Wbv[All Fields] OR low intensity vibration[All Fields] OR LIV[All Fields] OR VbX[All Fields] OR WBVT[All Fields] | 101846 |
| #9 | #8 OR #7 | 101846 |
| #10 | #3 AND #6 AND #9 | 663 |
| Cochrane Central Register of Controlled Trials | | |
| #1 | (sarcopeni* OR muscular atrophy OR muscle weakness OR fat free mass OR lean body mass OR lean mass OR body composition OR grip strength OR physical function OR Anthropometry): ti,ab,kw | 34462 |
| #2 | (seniors OR elderly OR older OR aged OR aging):ti,ab,kw | 436387 |
| #3 | (vibration OR whole body vibration OR whole body vibration training OR vibration exercise OR vibration platform OR vibratory therapy OR vibratory Plate OR sham therapy OR Wbv OR low intensity vibration OR LIV OR VbX OR WBVT): ti, ab,kw | 8108 |
| #4 | #1 AND #2 AND #3 | 376 |
|  | Trials matching on '"#4 - #1 AND #2 AND #3"' | 364 |
| EMBASE（ovid） | | |
| #1 | sarcopenia.mp. or exp muscle atrophy/ or exp muscle strength/ or exp sarcopenia/ or exp muscle mass/ or exp physical activity/ or exp body composition/ | 479207 |
| #2 | muscle weakness.mp. or exp muscle weakness/ | 260380 |
| #3 | exp lean body weight/ or fat free mass.mp. or exp fat free mass/ | 22573 |
| #4 | lean body mass.mp. | 8183 |
| #5 | muscle strength.mp. or exp muscle strength/ | 56444 |
| #6 | 1 or 2 or 3 or 4 or 5 | 723539 |
| #7 | aged/ | 2227543 |
| #8 | aging.mp. or exp aging/ | 423863 |
| #9 | 7 or 8 | 2553629 |
| #10 | exp vibration/ or exp whole body vibration/ or vibration.mp. | 69266 |
| #11 | vibratory therapy.mp. | 11 |
| #12 | sham therapy.mp. | 300 |
| #13 | Wbv.mp. | 1441 |
| #14 | low intensity vibration.mp. | 61 |
| #15 | VbX.mp. | 24 |
| #16 | WBVT.mp. | 65 |
| #17 | 10 or 11 or 12 or 13 or 14 or 15 or 16 | 69963 |
| #18 | 6 and 9 and 17 | 758 |
| CINAHL（EBhost） | | |
| #1 | mh(sarcopenia OR muscular atrophy OR muscle weakness ) | 7274 |
| #2 | AB sarcopeni* OR muscular atrophy OR muscle weakness OR fat free mass OR lean body mass OR lean mass OR body composition OR grip strength OR physical function OR Anthropometry | 42712 |
| #3 | S1 OR S2 | 47404 |
| #4 | mh(aged OR aging OR middle aged) | 705483 |
| #5 | AB seniors OR elderly OR older OR aged OR aging | 290316 |
| #6 | S4 OR S5 | 861842 |
| #7 | mh vibration | 3484 |
| #8 | AB vibration OR whole body vibration OR whole body vibration training OR vibration exercise OR vibration platform OR vibratory therapy OR vibratory Plate OR sham therapy OR Wbv OR low intensity vibration OR LIV OR VbX OR WBVT | 4721 |
| #9 | S7 OR S8 | 6602 |
| #10 | S3 AND S6 AND S9 | 123 |
| PEDro | | |
| #1 | sarcopeni* AND fitness training | 27 |
| #2 | sarcopeni* AND strength training | 37 |

| Study | Clearly stated aim | Inclusion of consecutive patients | Prospective collection of data | Endpoints appropriate to the aim of the study | Unbiased assessment of the study endpoint | Follow‐up period appropriate to the aim of the study | Loss to follow up less than 5% | Prospective calculation of the study size | Total score |
| --- | --- | --- | --- | --- | --- | --- | --- | --- | --- |
| Pietrangelo et al.  2009 | 2 | 2 | 2 | 2 | 0 | 1 | 2 | 0 | 11 |
| Shu fang et al.  2018 | 2 | 2 | 2 | 2 | 0 | 1 | 2 | 2 | 13 |
| Miller et al.  2018 | 2 | 2 | 2 | 2 | 1 | 2 | 0 | 0 | 11 |

Supplementary Table 2: MINORS scores of quasi-experimental studies
